# Supplementary material for: Oligodendrocytic Na+-K+-Cl– co-transporter 1 activity facilitates axonal conduction and restores plasticity in the adult mouse brain
Source: Nat Commun. 2021 Aug 26;12:5146. doi: 10.1038/s41467-021-25488-5 (PMC8390751; doi:10.1038/s41467-021-25488-5)
Supplement: Supplementary file 1 — Supplementary Information [file 41467_2021_25488_MOESM1_ESM.pdf]

Supplementary Information for

Oligodendrocytic Na^{+}-K^{+}-Cl^{-} co-transporter 1 activity facilitates axonal conduction and restores plasticity in the adult mouse brain

Yoshihiko Yamazaki[1*], Yoshifumi Abe[2], Satoshi Fujii[1], Kenji F. Tanaka[2]

[1]Department of Physiology, Yamagata University School of Medicine, Yamagata 990-9585, Japan

[2]Department of Neuropsychiatry, Keio University School of Medicine, Tokyo 160-8582, Japan

* Corresponding author. E-mail: yyamazak@med.id.yamagata-u.ac.jp

This PDF file includes:

Supplementary Figures

Supplementary Figure 1: *Nkcc1* expression on OL lineage cells.

Supplementary Figure 2: ChR2 is not expressed on premyelinating (newly formed) OLs.

Supplementary Figure 3: Expression of OL lineage markers and *Nkcc1* in wild-type and PLP-ChR2 mice.

Supplementary Figure 4: OL-mediated plasticity of axonal conduction is significant in juvenile mice.

Supplementary Figure 5: OL-mediated plasticity of conduction velocity is significant in juvenile mice.

Supplementary Figure 6: GABA-induced currents in OLs differ with postnatal development.

Supplementary Figure 7: *Nkcc1* overexpression in OLs does not affect OL development.

Supplementary Figure 8: Characterization of OLs in PLP-ChR2 mice overexpressing *Nkcc1*.

Supplementary Figure 9: *Nkcc1* overexpression facilitates OL-mediated plasticity of axonal conduction in adult mice.

Supplementary Figure 10: *Nkcc1* knockdown does not affect OL development.

Supplementary Figure 11: Characterization of OLs in *Nkcc1* knockdown mice.

Supplementary Tables

Supplementary Table 1: PCR primer sets used for mouse genotyping.

Supplementary Table 2: List of used antibodies.

Supplementary Discussion

Supplementary References

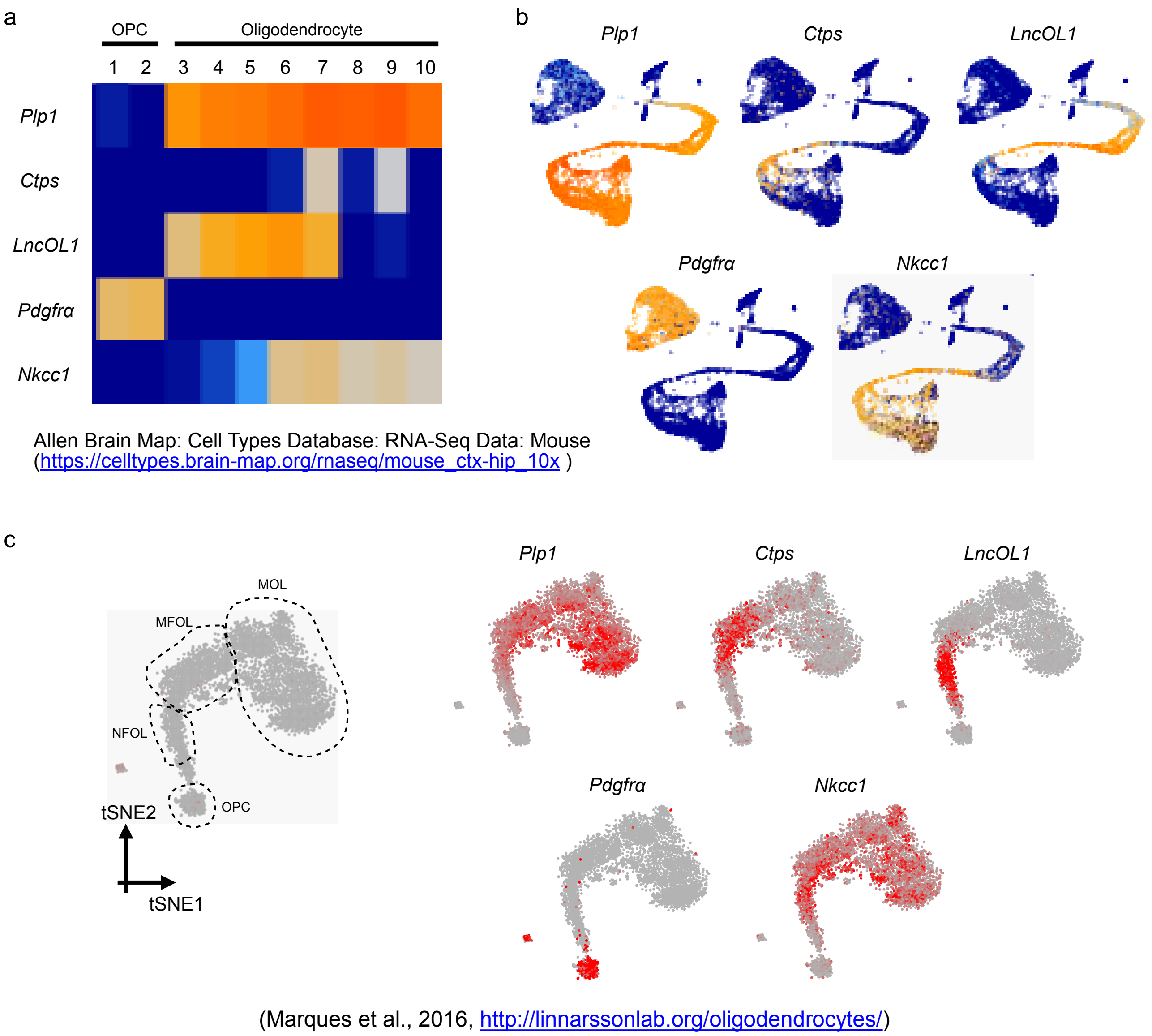

Supplementary Figure 1: *Nkcc1* expression on OL lineage cells.

a, Single cell RNA-sequencing showing the expression levels of *Pdgfrα*, *LncOL1* (also known as *9630013A20Rik*), *Ctps*, *Plp1*, and *Nkcc1* mRNA on OL precursor cells and OLs in adult mice. **b**, t-Distributed stochastic neighbor embedding (tSNE) analysis of data (**a**) showing that *Nkcc1* (also known as *Slc12a2*) is not expressed on OL precursor cells in adult mice. These data (**a** and **b**) were obtained from the Allen Brain Map: Cell Types Database: RNA-Seq Data: Mouse (https://celltypes.brain-map.org/rnaseq/mouse_ctx-hip_10x). **c**, tSNE analysis of single cell RNA-sequencing data showing *Pdgfrα*, *LncOL1*, *Ctps*, *Plp1*, and *Nkcc1* mRNA expression in mice at postnatal day 21, 30, and 60. These data were obtained from Marques et al., 2016 (http://linnarssonlab.org/oligodendrocytes/).

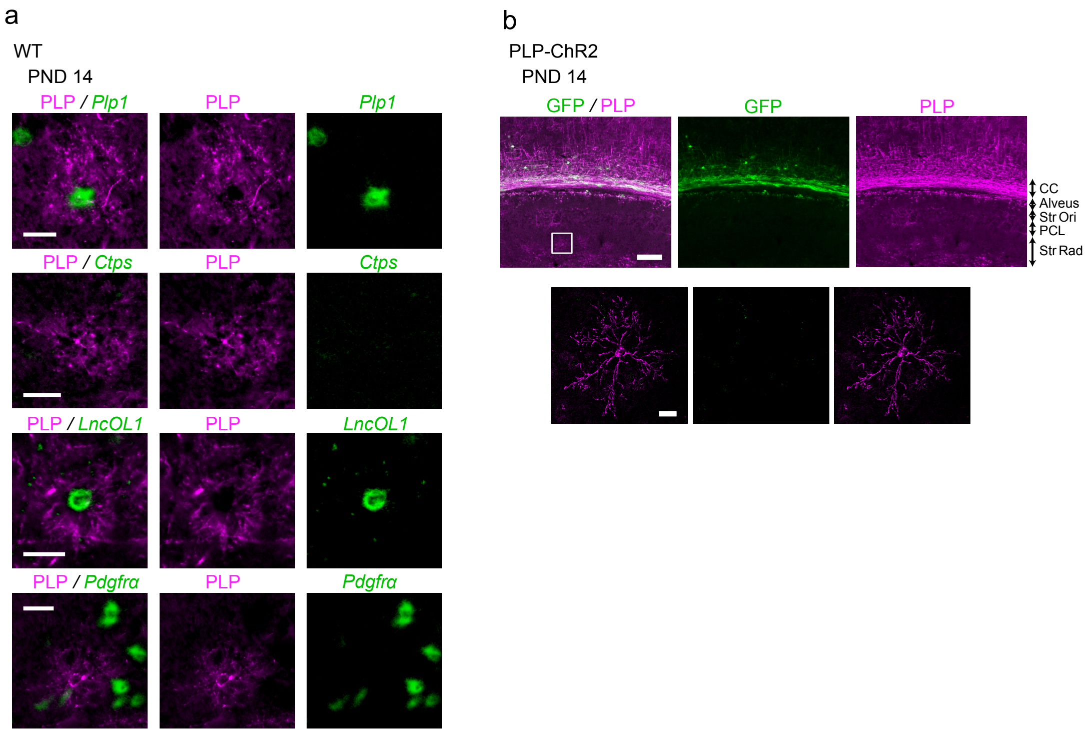

Supplementary Figure 2: ChR2 is not expressed on premyelinating OLs (newly formed OLs).

a, Hybrid staining of mRNA for each OL stage marker and PLP protein in wild-type (WT) mice at PND 14. DM20, an isoform of PLP protein, was overlaid with *LncOL1*-positive cells. DM20 was identified by its radial morphology. Scale bars, 30 μm. Similar results were observed in three mice. **b**, EYFP-expressing cells were not co-labeled with DM20 in PLP-ChR2 mice at PND 14. Scale bars, 100 and 10 μm for lower and higher magnification images, respectively. Similar results were observed in three mice. PND in this and following figures indicates postnatal day.

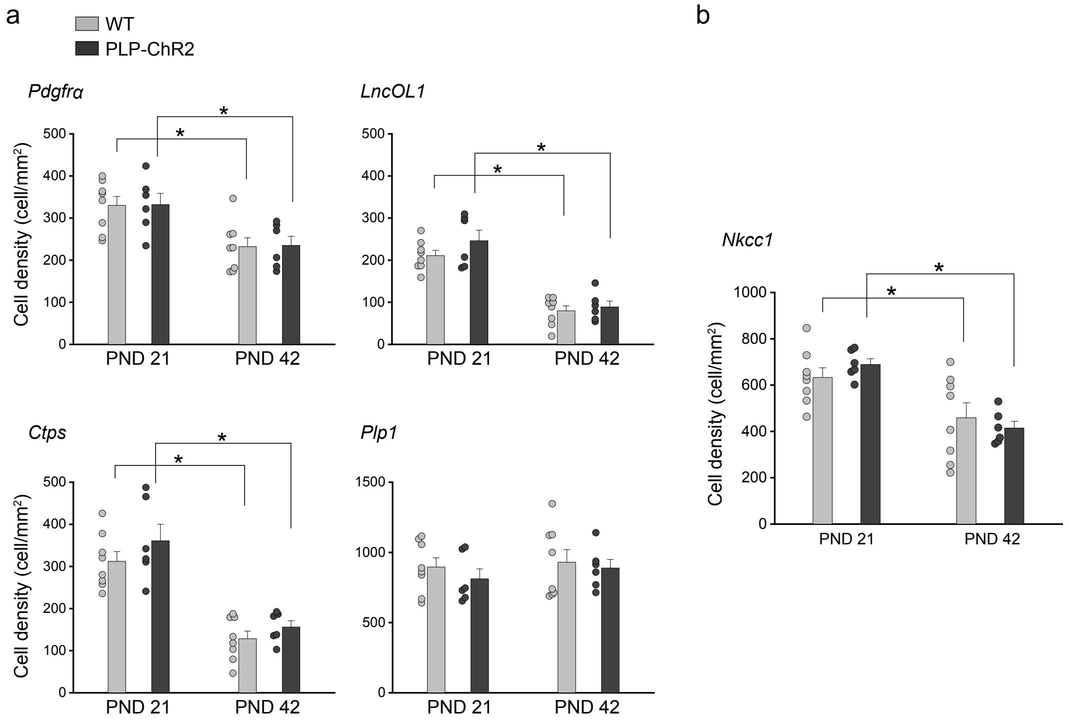

Supplementary Figure 3: Expression of OL lineage markers and *Nkcc1* in wild-type and PLP-ChR2 mice.

a, Expression of *Pdgfrα*, *LncOL1*, *Ctps*, and *Plp1* mRNA in wild-type (WT) and PLP-ChR2 mice at PND 21 and 42. The cells were counted in the area including the corpus callosum, alveus, and stratum oriens. Data were obtained from 8 slices from 4 WT mice and from 6 slices from 3 PLP-ChR2 mice for each gene. Unpaired two-sided Student's t-test (PND 21 vs PND 42), *Pdgfrα* in WT, $t_{14} = 3.30$, $P = 0.0052$; *Pdgfrα* in PLP-ChR2, $t_{10} = 2.81$, $P = 0.018$; *LncOL1* in WT, $t_{14} = 7.64$, $P < 0.001$; *LncOL1* in PLP-ChR2, $t_{10} = 5.53$, $P < 0.001$; *Ctps* in WT, $t_{14} = 6.26$, $P < 0.001$; *Ctps* in PLP-ChR2, $t_{10} = 4.89$, $P = 0.0023$. Data are presented as mean ± SEM. *$P < 0.05$. **b**, Developmental changes in the expression of *Nkcc1* mRNA in the hippocampus of WT and PLP-ChR2 mice. The cells were counted in the area including the corpus callosum, alveus, and stratum oriens (n = 8 slices from 4 WT mice and n = 6 from 3 PLP-ChR2 mice). Unpaired two-sided Student's t-test, PND 21 vs PND 42 in WT, $t_{14} = 2.27$, $P = 0.040$; in PLP-ChR2, $t_{10} = 7.20$, $P < 0.001$. Data are presented as mean ± SEM. *$P < 0.05$.

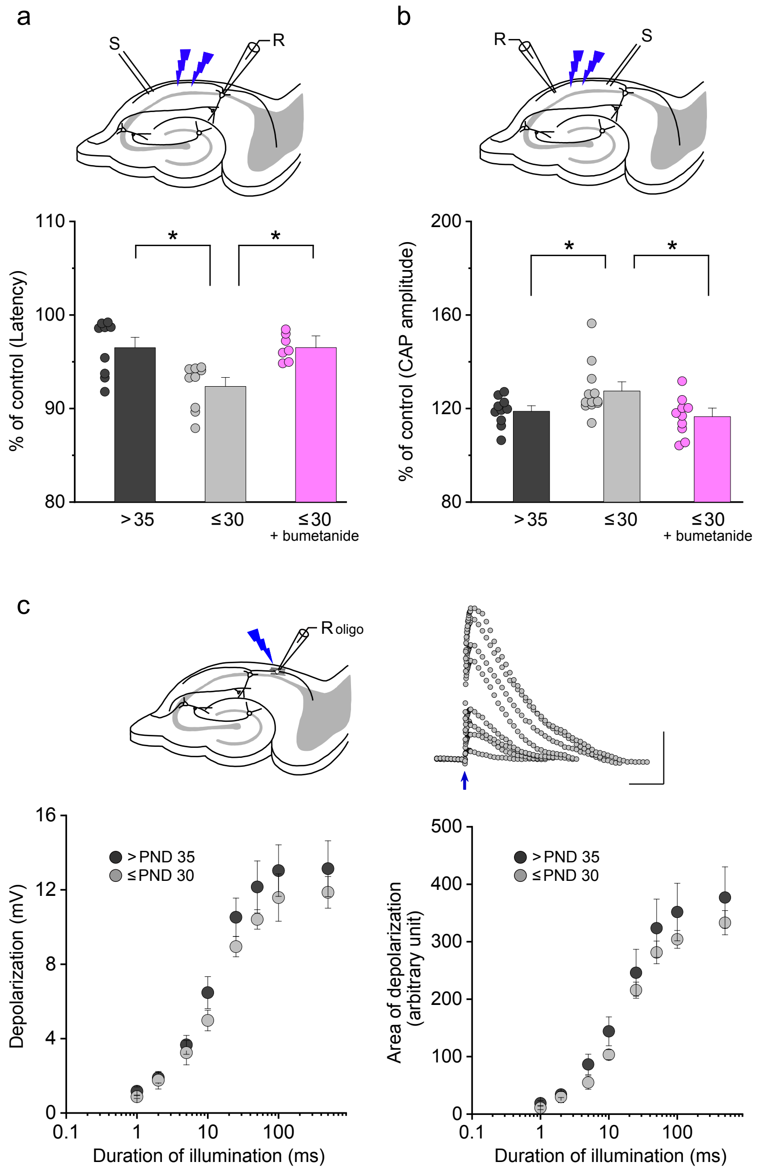

Supplementary Figure 4: OL-mediated plasticity of axonal conduction is significant in juvenile mice.

a, Recording of antidromic action potentials in a CA1 pyramidal cell. Summary histogram for the changes in the latency of action potentials along the axons induced by OL depolarization from Fig. 2c. Unpaired two-sided Student's t-test, ≤PND 30 vs >PND 35: t_{16} = 3.23, P = 0.0052; ≤PND 30 vs ≤PND 30 in the presence of bumetanide: t_{14} = 3.96, P = 0.0014. Data are presented as mean ± SEM. *P < 0.05. **b**, Recording of compound action potentials (CAPs) in the alveus. Summary histograms for the change in CAP amplitude from Fig. 2d. Unpaired two-sided Student's t-test, ≤PND 30 vs >PND 35: t_{20} = 2.18, P = 0.042; ≤PND 30 vs ≤PND 30 in the presence of bumetanide: t_{20} = 2.55, P = 0.019. Data are presented as mean ± SEM. *P < 0.05. **c**, Recording of depolarizing responses from a current-clamped OL in the alveus by blue light photostimulation of different durations (1, 2, 5, 10, 25, 50, 100, or 500 ms). Scale, 100 s and 5 mV. Depolarization peak and area of the depolarization curve plotted against the duration of illumination in younger PLP-ChR2 mice (≤PND 30 [22–24], n = 3, using 3 slices from 3 animals) and older mice (>PND 35 [36–43], n = 4, using 4 slices from 4 animals). Data are presented as mean ± SEM. *P < 0.05.

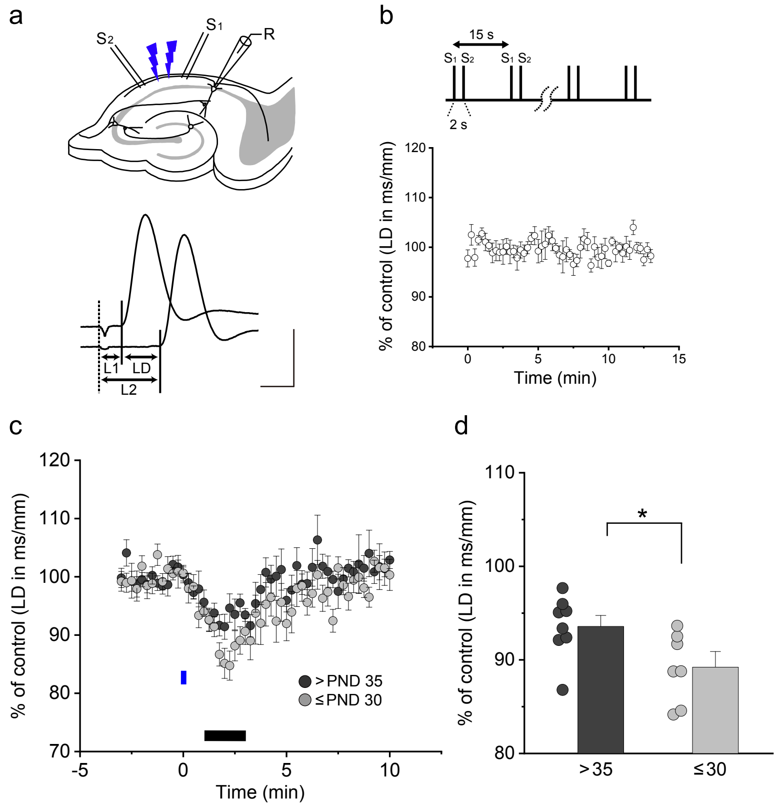

Supplementary Figure 5: OL-mediated plasticity of conduction velocity is significant in juvenile mice.

a, Recording of antidromic action potentials in CA1 pyramidal cells at two distinct positions and measurement of the conduction latency difference (LD; L2-L1). Scale, 2 ms, 50 mV. **b**, Electrical stimulation protocol of alternative stimulation at S_1 and S_2 to obtain LD. Time-course of the LDs (in ms/mm) of action potentials during alternative stimulation, showing that alternative stimulation itself does not affect axonal conduction velocity (n = 6). Data are presented as mean ± SEM. **c**, Time-course of the LDs after photostimulation in PLP-ChR2 mice at >PND 35 (n = 8, PND 36–38), and ≤PND 30 (n = 7, PND 27–29). The linear distance between the two stimulating electrodes was kept as constant as possible to obtain similar LDs (0.72 ± 0.11 ms, 1.69 ± 0.20 ms/mm for >PND 35 and 0.64 ± 0.11 ms, 1.57 ± 0.18 ms/mm for ≤PND 30). Data are presented as mean ± SEM. **d**, Summary histogram for the changes in LDs along the axons induced by OL depolarization. Unpaired two-sided Student's t-test, t_{13} = 2.42, P = 0.030. Data are presented as mean ± SEM. *P < 0.05.

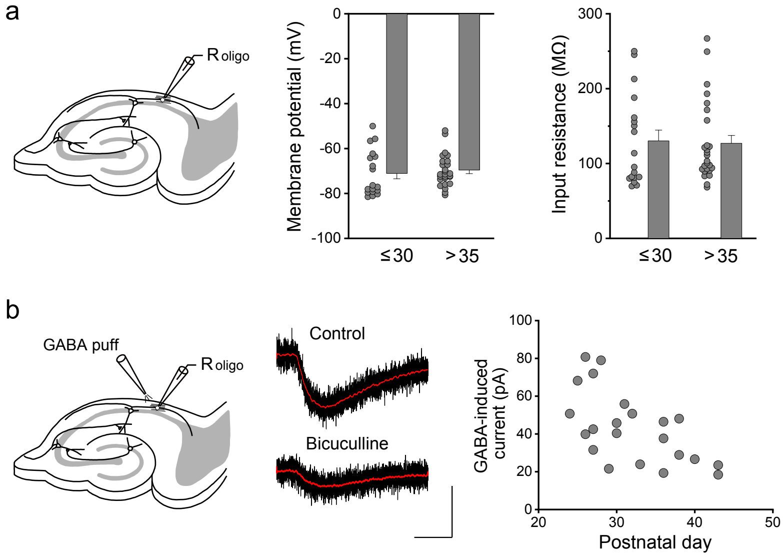

Supplementary Figure 6: GABA-induced currents in OLs differ with postnatal development.

a, Schematic drawing showing the recording electrode (R oligo) for an OL in the alveus. Resting membrane potential and input resistance of OLs in younger mice (≤PND] 30 [15–30], *n* = 18) and older mice (>PND 35 [35–46], *n* = 26). Data are presented as mean ± SEM. **b**, Schematic drawing showing the recording (R oligo) and GABA puffer pipettes. GABA-induced currents in the absence and presence of bicuculline. Scale, 2 s and 50 pA. Relationship between PND and the magnitude of GABA-induced inward currents (*n* = 22).

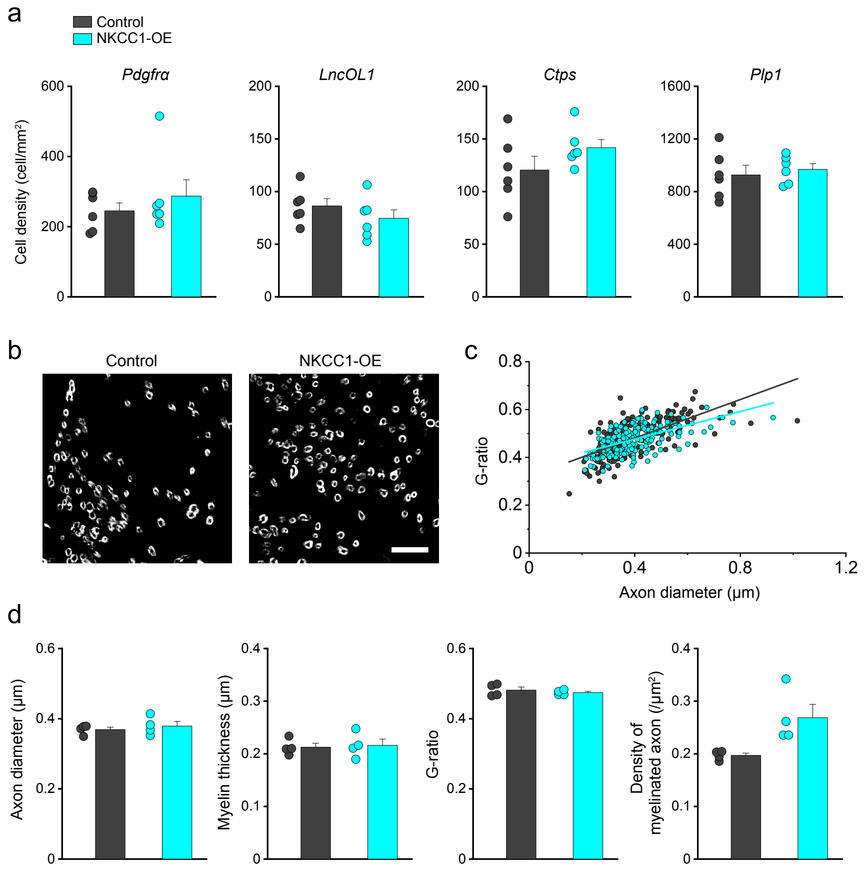

Supplementary Figure 7: *Nkcc1* overexpression in OLs does not affect OL development.

a, Expression of *Pdgfra*, *LncOL1*, *Ctps*, and *Plp1* mRNA in mice overexpressing *Nkcc1* (NKCC1-OE) at PND 42. The cells were counted in the area including the corpus callosum, alveus, and stratum oriens (*n* = 6 slices from 3 animals of each gene). Data are presented as mean ± SEM. **b**, Super resolution micrographs from the alveus of control and NKCC1-OE mice at PND 42. Scale bar, 5 µm. Similar results were observed in four mice in each group. **c**, Relationship between axon diameter and g-ratio in myelinated axons in the alveus. **d**, Group data of axon diameter, myelin thickness, and g-ratio in the alveus, and density of myelinated axons in the stratum oriens of control mice (*n* = 4) and NKCC1-OE mice (*n* = 4) at PND 42. Data are presented as mean ± SEM.

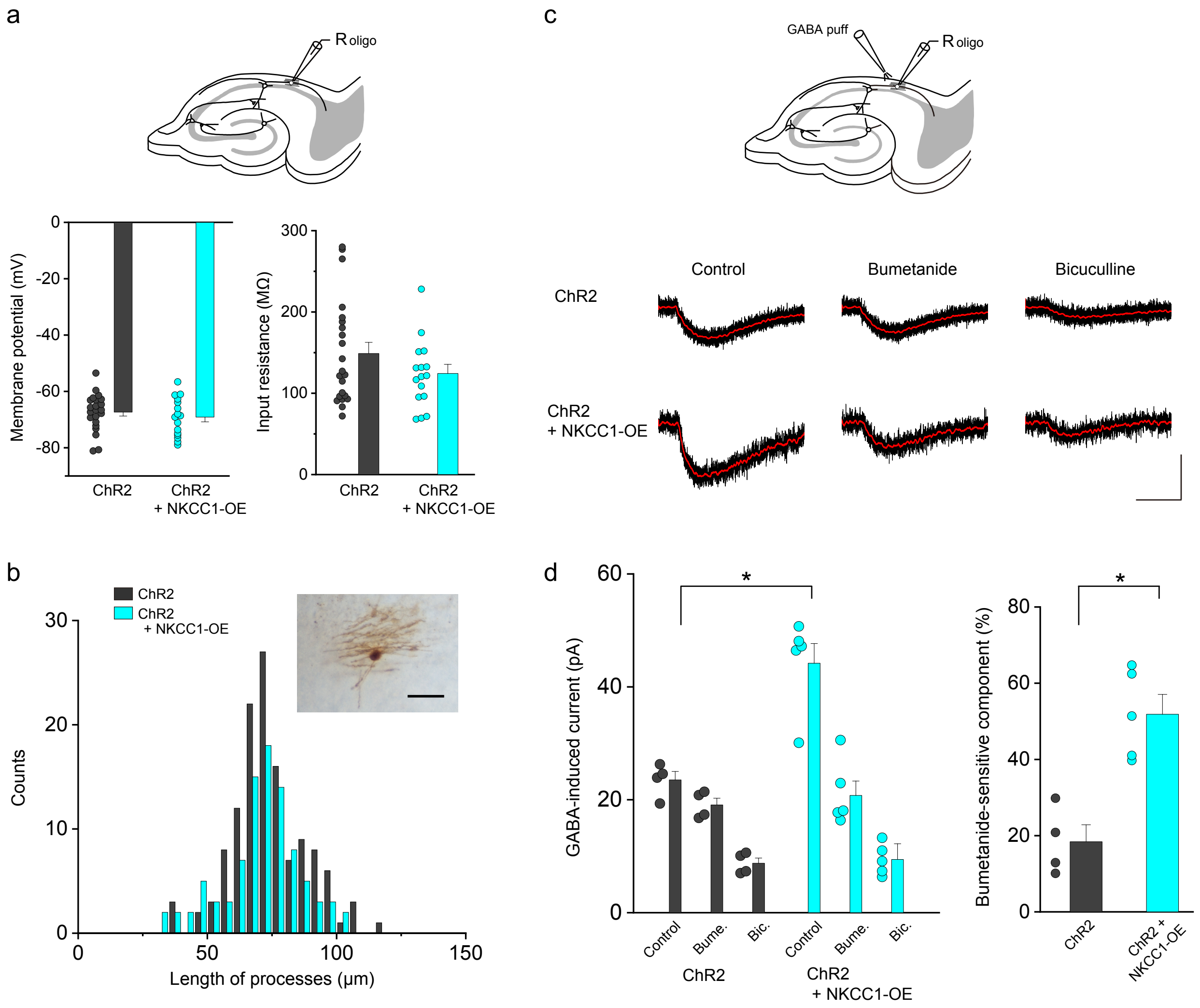

Supplementary Figure 8: Characterization of OLs in PLP-ChR2 mice overexpressing *Nkcc1*.

a, Resting membrane potential and input resistance of OLs in PLP-ChR2 mice (n = 22, PND 36–46) or PLP-ChR2 mice overexpressing *Nkcc1* (NKCC1-OE, n = 16, PND 37–48). Data are presented as mean ± SEM. **b**, Distribution of the length of OL processes in PLP-ChR2 mice (n = 128 from 10 cells, PND 36–44) and PLP-ChR2 mice overexpressing *Nkcc1* (n = 92 from 7 cells, PND 36–46). Inset is a light microscopy image showing a biocytin-stained OL in a PLP-ChR2 mouse overexpressing *Nkcc1*. Scale, 50 μm. **c**, Schematic drawing showing the recording pipette (R_{oligo}), GABA puffer pipette, and typical GABA-induced inward currents in the absence and presence of bumetanide and bumetanide plus bicuculline. Scale, 2 s and 40 pA. **d**, Summary histograms for the magnitude of GABA-induced inward currents and the effects of bumetanide and bicuculline on the currents in PLP-ChR2 mice (n = 4, PND 37–44) or PLP-ChR2 mice overexpressing *Nkcc1* (n = 5, PND 36–43). Unpaired two-sided Student's t-test, magnitude: t_7 = 4.81, P = 0.0019; bumetanide-sensitive component: t_7 = 4.73, P = 0.0021. Data are presented as mean ± SEM. *P < 0.05.

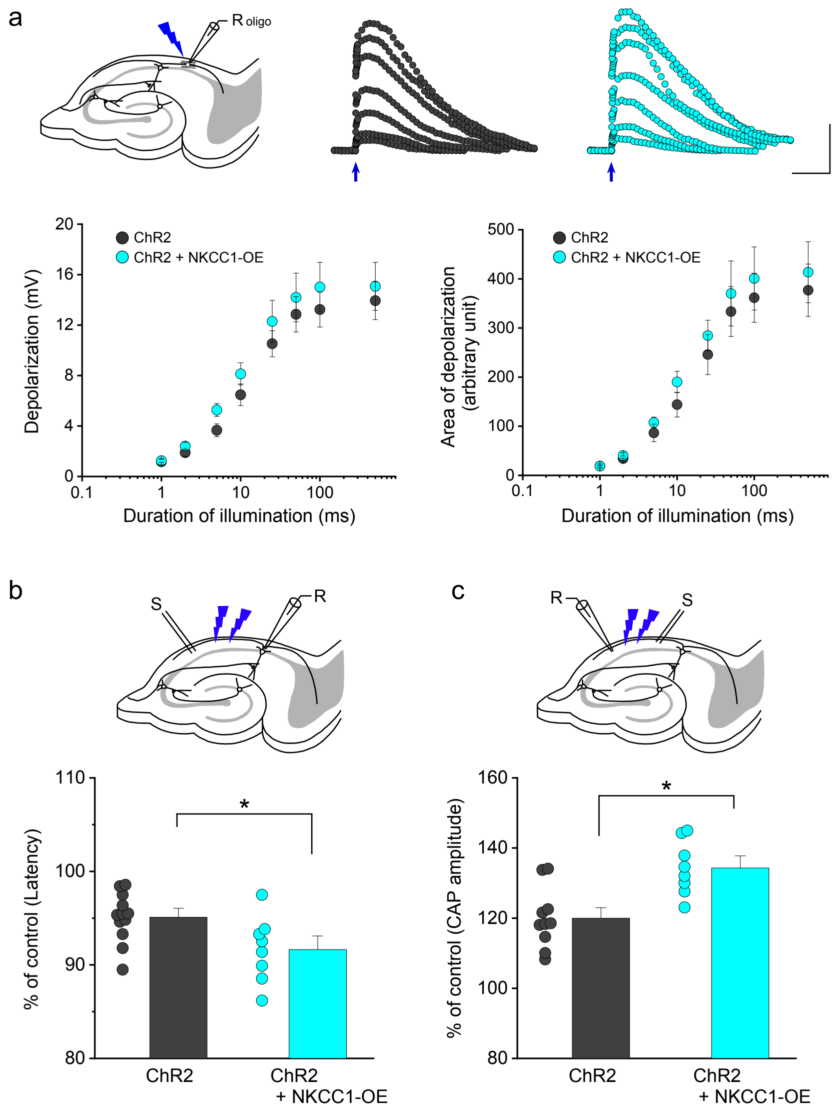

Supplementary Figure 9: *Nkcc1* overexpression facilitates the OL-mediated plasticity of axonal conduction in adult mice.

a, Recording of the depolarizing responses from a current-clamped OL in the alveus by blue light photostimulation of different durations (1, 2, 5, 10, 25, 50, 100, or 500 ms) in PLP-ChR2 mice and PLP-ChR2 mice overexpressing *Nkcc1* (NKCC1-OE). Scale, 100 s and 5 mV. Depolarization peak and area of the depolarization curve plotted against the duration of illumination in PLP-ChR2 mice (n = 4, using 4 slices from 4 animals, PND 36–43) and PLP-ChR2 mice overexpressing *Nkcc1* (n = 5, using 5 slices from 4 animals, PND 35–44). The data from PLP-ChR2 mice (dark gray dots) correspond with the data from >PND 35 mice shown in Supplementary Fig. 4a. Data are presented as mean ± SEM. **b**, Recording of antidromic action potentials in a CA1 pyramidal cell. Summary histogram for the changes in the latency of action potentials by OL depolarization from Fig. 4c (PLP-ChR2 mice: n = 12; PLP-ChR2 mice overexpressing Nkcc1: n = 8). Unpaired two-sided Student's t-test, t_{18} = 2.11, P = 0.048. Data are presented as mean ± SEM. *P < 0.05. **c**, Recording of compound action potentials (CAPs) in the alveus. Summary histogram for the change in CAP amplitude after photostimulation from Fig. 4d. (PLP-ChR2 mice: n = 10; PLP-ChR2 mice overexpressing Nkcc1: n = 8). Unpaired two-sided Student's t-test, t_{16} = 3.65, P = 0.0022. Data are presented as mean ± SEM. *P < 0.05.

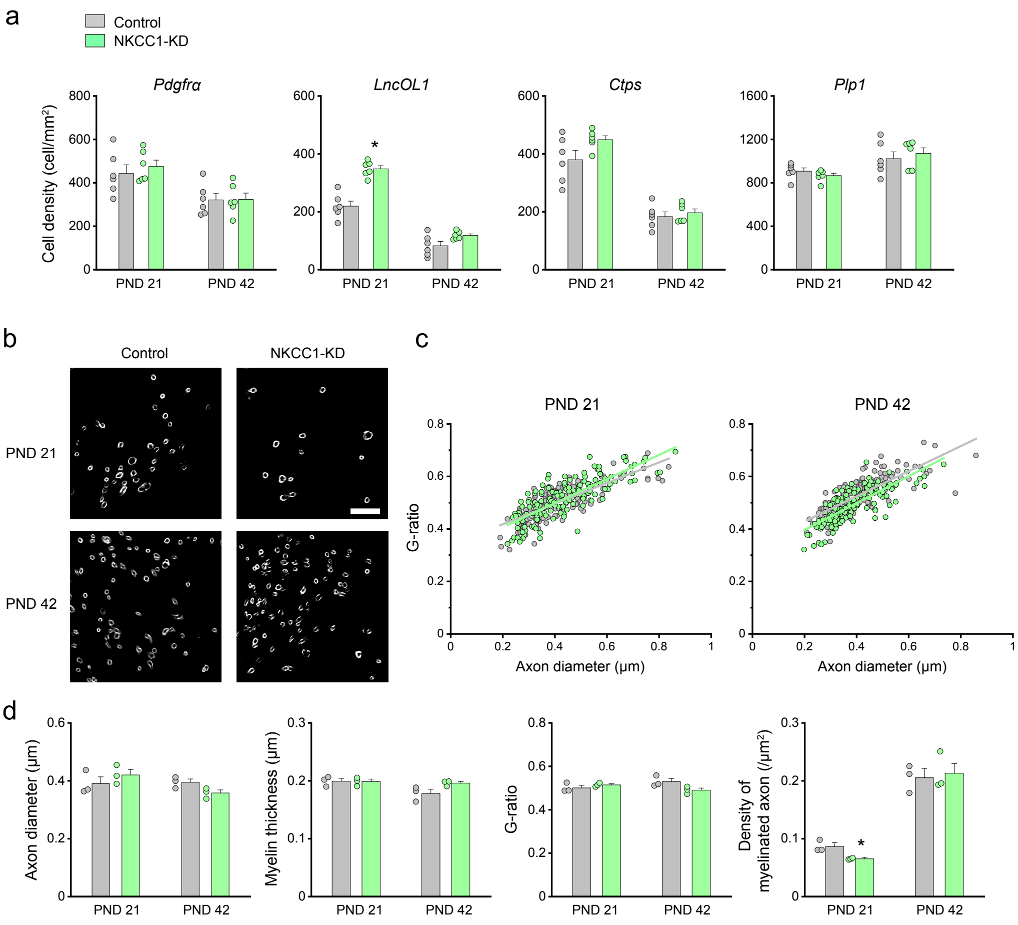

Supplementary Figure 10: *Nkcc1* knockdown does not affect OL development.

a, Cell counts for *Pdgfrα*-, *LncOL1*-, *Ctps*-, and *Plp1*-positive cells in the area including the corpus callosum, alveus, and stratum oriens (n = 6 slices from 3 animals) in control mice and *Nkcc1* knockdown (NKCC1-KD) mice at PND 21 and 42. Unpaired two-sided Student's t-test, t_{10} = 6.33, P < 0.001. Data are presented as mean ± SEM. *P < 0.05. **b**, Super-resolution micrographs from control and NKCC1-KD mice at PND 21 and 42. Scale bar, 5 μm. Similar results were observed in three mice in each group. **c**, Relationship between axon diameter and g-ratio in myelinated axons in the alveus. **d**, Group data of axon diameter, myelin thickness, and g-ratio in the alveus, and density of myelinated axons in the stratum oriens of control and NKCC1-KD mice at PND 21 and 42 (n = 3 each). Unpaired two-sided Student's t-test, t_{10} = 2.97, P = 0.022. Data are presented as mean ± SEM. *P < 0.05.

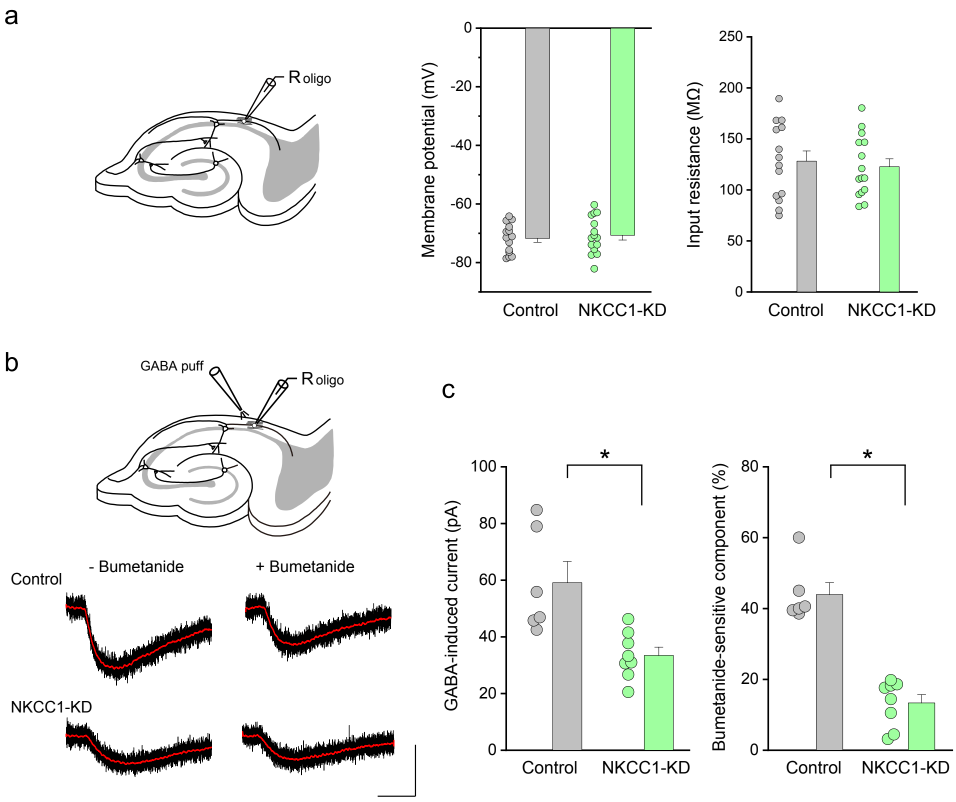

Supplementary Figure 11: Characterization of OLs in *Nkcc1* knockdown mice.

a, Resting membrane potential and input resistance of OLs in control mice (*n* = 14, PND 25–30) or *Nkcc1* knockdown (NKCC1-KD) mice (*n* = 15, PND 24–30). Data are presented as mean ± SEM. **b**, Schematic drawing showing the recording pipette (R $_{\text{oligo}}$), GABA puffer pipette, and typical GABA-induced inward currents in the absence and presence of bumetanide. Scale, 2 s and 40 pA. **c**, Summary histograms for the magnitude of GABA-induced inward currents and the effects of bumetanide on the currents in control mice (*n* = 6, PND 25–30) or NKCC1-KD mice (*n* = 8, PND 27–30). Unpaired two-sided Student's t-test, magnitude: t_{12} = 3.56, *P* = 0.0039; bumetanide-sensitive component: t_{12} = 7.77, *P* < 0.001. Data are presented as mean ± SEM. **P* < 0.05.

Primer name	Sequence	Use
tetO intron (forward)<br>tetO up (reverse)	AAGGCAGGATGATGACCAGGATGT<br>AGCAGAGCTCGTTTAGTGAACCGTT	Genotype tetO-ChR2(C128S)-YFP mouse
PLPU-604 (forward)<br>mtTA24L (reverse)	TTTCCCATGGTCTCCCTTGAGCTT<br>CGGAGTTGATCACCTTGGACTTGT	Genotype PLP-tTA mouse
ttsP1 (forward)<br>ttsP2 (reverse)	TTGATCACCAAGGTGCAGAG<br>CAGGGCTCTTCTCCCTTCTC	Genotype Actin-tTS mouse
NKCC142L (forward)<br>NKCC-136U (reverse)	TCCCAGCCGTAGTAGCATCCTCTT<br>TGTAGTGGCGCTGTGACTCTTTCT	Genotype Nkcc1$^{tetO/tetO}$ mouse
tetO up (forward)<br>NKCC-136U (reverse)	AGCAGAGCTCGTTTAGTGAACCGTT<br>TGTAGTGGCGCTGTGACTCTTTCT	Genotype Nkcc1$^{tetO/+}$ mouse

Supplementary Table 1: PCR primer sets used for mouse genotyping.

Antibody name	Supplier name	Catalog number	Lot number
Rat monoclonal PLP antibody (clone AA3)	Yamamura et al.[1]	N.A.	N.A.
Goat polyclonal GFP antibody	Rockland Immunochemicals	600-101-215	34589
Rabbit polyclonal NG2 antibody	Millipore	AB5320	3422864
Donkey anti-rat antibody-Alexa 594 conjugated	Invitrogen	A21209	1905801
Donkey anti-rat antibody-Alexa647 conjugated	Abcam	ab150155	GR3285492-1
Donkey anti-rabbit antibody-Alexa488 conjugated	Invitrogen	A21206	1480470
Donkey anti-rabbit antibody-Alexa555 conjugated	Invitrogen	A31572	1945911
Donkey anti-goat antibody-Alexa488 conjugated	Invitrogen	A32814	VA293145
Alkaline phosphatase-conjugated anti-DIG antibody	Roche	11093274910	32871922
Peroxidase-conjugated anti-FITC antibody	Roche	11426346910	45220020

Supplementary Table 2: List of used antibodies.

Supplementary Discussion

Myelinated fiber plasticity, as described in this study, includes an increase in conduction velocity and an enhancement of axonal excitability. Conduction velocity along myelinated axons is influenced by axonal (diameter and axoplasmic conductance), nodal (area, capacitance, and conductance), and internodal (intermodal length and myelin capacitance) parameters[2]. The increase in conduction velocity induced by oligodendrocyte (OL) depolarization is very likely due to a morphological change in OL processes.

Since ChR2 is non-selective cation channel, the activation of ChR2 and subsequent activation of voltage-dependent ion channels induce cation influx. In physiological conditions, the activation of non-NMDA and NMDA receptors contributes to ion influx with depolarization by responding to glutamate released from neurons. As the intracellular volume of the myelinating processes is very small, ion influx across the plasma membrane leads to structural changes in the processes of OLs as a result of osmotic swelling. Structural changes in the myelinating processes influence the extent of insulation at the paranodal and internodal regions. As the periaxonal and paranodal submyelin spaces form a current pathway[3], axonal conduction along myelinated axons is sensitive to morphological changes at OL processes. When the paranodal loops swell, they may wrap the axons more tightly and the insulation at the paranodal region may increase, resulting in more effective current flow from one node to the next[4]. Thus, the conduction velocity of action potentials would be increased. The contribution of NKCC1 activity to the increase in conduction velocity induced by OL depolarization strongly supports the notion that the morphological change by osmotic alteration is a probable mechanism for myelinated fiber plasticity. Since the increase in conduction velocity appears within a few minutes after OL depolarization and is sustained for approximately 10 min, it is unlikely that *de novo* myelination and myelin remodeling are involved in the plastic changes of conduction velocity.

Since OLs are depolarized in response to extracellular K^+ concentrations increased by neuronal activity, the involvement of K^+ channels in the observed axonal plasticity is suspected. Ba^{2+} and 4-AP (which act on different K^+ channels) both inhibit the plastic changes related to the increase in conduction velocity[5, 6]. While 4-AP has no significant effect on light-evoked OL depolarization, Ba^{2+} significantly suppresses depolarization. Thus, the changes in 4-AP-sensitive K^+ channels expressed on axons are likely involved in

the increase in conduction velocity. The suppressive effect of Ba^{2+} is probably due to the suppression of OL depolarization by photostimulation.

In the plasticity of axonal excitability, the amplitude and area of CAP are increased, while CAP width is unchanged. These changes in CAP parameters result from the increase in the number of firing axons by electrical stimulation and/or an increase in the amplitude and/or duration of each action potential conducted along each axon, but not from an increase in conduction velocity[5]. However, through analysis of the paired-pulse ratio at destination synapses, the changes in the kinetics of each action potential can be excluded[7]. Thus, the increase in axon excitability is due to a decrease in the threshold for the generation of action potentials.

It is clear that ion channel activation for action potential generation and the maintenance of the local ionic environment to allow for action potential propagation mainly contribute to axonal excitability[8]. The enhancement of axonal excitability is inhibited by the application of Ba^{2+}, but not by 4-AP. Since Ba^{2+} also significantly suppresses light-evoked OL depolarization, it is suggested that the inhibition of OL depolarization caused by Ba^{2+} application inhibits the enhancement of axonal excitability, and that the changes in the properties of Ba^{2+}-sensitive K^{+} channels on axons and/or OLs (as a result of OL depolarization) lead to the enhancement of axonal excitability[5].

In addition to the contribution of K^{+} channels, since the plasticity of axonal excitability occurs over a slower and longer timescale (beginning at several minutes after OL depolarization and lasting for more than 3 h), it could be related to a certain type of myelin remodeling. In myelinated axons, the extent of Na^{+} channel clustering[9] and the distribution of K^{+} channels[10] at the node affect the action potential threshold of myelinated axons. Therefore, the expression location and extent of clustering of ion channels at the node (which are affected by changes in nodal structure) would be involved in the plasticity of axonal excitability. The change in node length[11] or Ca^{2+}-dependent elongation of OL processes occurring within 1–2 h[12, 13], both of which are one form of myelin remodeling in response to neural activity, would be related to the OL depolarization-induced enhancement of axonal excitability.

Related to the mechanisms for increased conduction velocity and enhanced axonal excitability, we must mention the roles of neurotransmitters, since it is possible that OLs signal to axons using neuroactive substances and since axonal conduction is regulated by neurotransmitter-mediated mechanisms. Similarly, as OL depolarization decreases K^{+}

buffering, it is possible that the extracellular K^+ concentration around axons increases, resulting in increased conduction velocity and enhanced axonal excitability. However, pharmacological experiments applying various neurotransmitter receptor antagonists have not shown significant effects on axonal conduction[5]. Thus, it is unlikely that the release of neuroactive substances from depolarizing OLs is involved in myelinated fiber plasticity. Moreover, it has been confirmed that a transient increase in extracellular K^+ concentration itself does not contribute to myelinated fiber plasticity[5].

The magnitude of the plasticity of axonal excitability also varies depending on NKCC1 activity. Thus, OLs (in addition to their prevailing role in saltatory conduction) regulate axonal conduction plasticity in conjunction with changes in K^+ channels, probably through NKCC1-mediated morphological alterations which could be integrated into myelin plasticity. It is possible that structural myelin plasticity occludes the changes in the properties of ion channels.

Supplementary Rreferences

1. Yamamura, T. et al.Monoclonal Antibodies against myelin proteolipid protein: Identification and characterization of two major determinants. *J. Neurochem.* **57**, 1671–1680 (1991).

2. Ritchie JM. Physiology of axon. In: Waxman SG, Kocsis JD, Stys PK editors. The axon. New York: Oxford UP. pp 68–96 (1995).

3. Cohen, C. C. H. et al. Saltatory conduction along myelinated axons involves a periaxonal nanocircuit. *Cell* **180**, 311–322 (2020).

4. Yamazaki, Y. et al. Oligodendrocytes: facilitating axonal conduction by more than myelination. *Neuroscientist* **16**, 11–18 (2010).

5. Yamazaki, Y. et al. Short- and long-term functional plasticity of white matter induced by oligodendrocyte depolarization in the hippocampus. *Glia* **62**, 1299–1312 (2014).

6. Yamazaki, Y. Oligodendrocyte physiology modulating axonal excitability and nerve conduction. *Adv. Exp. Med. Biol.* **1190**, 123–144 (2019).

7. Yamazaki, Y. et al. Region- and cell type-specific facilitation of synaptic function at destination synapses induced by oligodendrocyte depolarization. *J. Neurosci.* **39**, 4036–4050 (2019).

8. Kiernan, M. C. & Kaji, R. Physiology and pathophysiology of myelinated nerve fibers. In: Said G, Krarup C (eds) Peripheral nerve disorders. Handbook of clinical neurology, vol 115. Elsevier, Amsterdam, pp 43–53 (2013).

9. Battefeld, A. et al. Heteromeric Kv7.2/7.3 channels differentially regulate action potential initiation and conduction in neocortical myelinated axons. *J. Neurosci.* **34**, 3719–3732 (2014).

10. Eshed-Eisenbach, Y. & Peles, E. The clustering of voltage-gated sodium channels in various excitable membranes. *Dev. Neurobiol.* doi: 10.1002/dneu.22728 (2019).

11. Chapman, T.W. & Hill, R. A. Myelin plasticity in adulthood and aging. *Neurosci. Lett.* **715**, 134645 (2020).

12. Baraban, M., Koudelka, S. & Lyons, D.A. Ca^{2+} activity signatures of myelin sheath formation and growth in vivo. *Nat. Neurosci* **21**, 19–23 (2018).

13. Krasnow, A. M. et al. Regulation of developing myelin sheath elongation by oligodendrocyte calcium transients in vivo. *Nat. Neurosci.* **21**, 24–28 (2018).
