# Supplementary material for: Oligodendrocytic Na+-K+-Cl– co-transporter 1 activity facilitates axonal conduction and restores plasticity in the adult mouse brain
Source: Nat Commun. 2021 Aug 26;12:5146. doi: 10.1038/s41467-021-25488-5 (PMC8390751; doi:10.1038/s41467-021-25488-5)
Supplement: Supplementary file 3 — Reporting Summary [file 41467_2021_25488_MOESM3_ESM.pdf]

nature research

Corresponding author(s): Yoshihiko Yamazaki

Last updated by author(s): Jul 30, 2021

Reporting Summary

Nature Research wishes to improve the reproducibility of the work that we publish. This form provides structure for consistency and transparency in reporting. For further information on Nature Research policies, see our Editorial Policies and the Editorial Policy Checklist.

Statistics

For all statistical analyses, confirm that the following items are present in the figure legend, table legend, main text, or Methods section.

n/a	Confirmed	
☐	☒	The exact sample size (n) for each experimental group/condition, given as a discrete number and unit of measurement
☐	☒	A statement on whether measurements were taken from distinct samples or whether the same sample was measured repeatedly
☐	☒	The statistical test(s) used AND whether they are one- or two-sided<br>*Only common tests should be described solely by name; describe more complex techniques in the Methods section.*
☐	☒	A description of all covariates tested
☐	☒	A description of any assumptions or corrections, such as tests of normality and adjustment for multiple comparisons
☐	☒	A full description of the statistical parameters including central tendency (e.g. means) or other basic estimates (e.g. regression coefficient) AND variation (e.g. standard deviation) or associated estimates of uncertainty (e.g. confidence intervals)
☐	☒	For null hypothesis testing, the test statistic (e.g. F, t, r) with confidence intervals, effect sizes, degrees of freedom and P value noted<br>*Give P values as exact values whenever suitable.*
☒	☐	For Bayesian analysis, information on the choice of priors and Markov chain Monte Carlo settings
☒	☐	For hierarchical and complex designs, identification of the appropriate level for tests and full reporting of outcomes
☒	☐	Estimates of effect sizes (e.g. Cohen's d, Pearson's r), indicating how they were calculated

Our web collection on statistics for biologists contains articles on many of the points above.

Software and code

Policy information about availability of computer code

Data collection	Each data collection procedures are described in the Methods. Electrophysiological signals were collected with data acquisition program developed by ourselves using Visual Basic 6.0. Any data collection programs are available upon reasonable request.
Data analysis	Data were analyzed with data analysis program developed by ourselves using Visual Basic 6.0, MATLAB 2018b (in-house code), Origin 2020b and mage J v1. 53k. Scripts used for data analysis are available upon reasonable request.

For manuscripts utilizing custom algorithms or software that are central to the research but not yet described in published literature, software must be made available to editors and reviewers. We strongly encourage code deposition in a community repository (e.g. GitHub). See the Nature Research guidelines for submitting code & software for further information.

Data

Policy information about availability of data

All manuscripts must include a data availability statement. This statement should provide the following information, where applicable:

- Accession codes, unique identifiers, or web links for publicly available datasets
- A list of figures that have associated raw data
- A description of any restrictions on data availability

The datasets generated and analyzed in the current study are available from the corresponding author upon reasonable request. The data underlying the figures are provided as a Source Data file. Source data are provided with this paper.

Field-specific reporting

Please select the one below that is the best fit for your research. If you are not sure, read the appropriate sections before making your selection.

- [x] Life sciences
- [ ] Behavioural & social sciences
- [ ] Ecological, evolutionary & environmental sciences

For a reference copy of the document with all sections, see nature.com/documents/nr-reporting-summary-flat.pdf

Life sciences study design

All studies must disclose on these points even when the disclosure is negative.

Sample size	No statistical methods were used to predetermine sample sizes. Sample size was determined based on previous experience, standards in the field, and our technical ability to maximize sample size. Sample sizes are noted in each case.
Data exclusions	Only experimental sessions that were prematurely terminated due to major technical failures and/or instability recording condition were excluded from analysis. As stated in Methods, whole cell-recordings were accepted if series resistance varied by <25%. This was based on previous studies on topics of axonal conduction plasticity and synaptic plasticity and was necessary for accurate measurement of conduction velocity and synaptic strength (Yamazaki et al., Glia, 2014; Yamazakai et al., J Neurosci, 2019).
Replication	Individual experiments were performed either in duplicate or triplicate, and experiments were independently repeated at least three times. Standard statistical methods were used to accept or reject null hypotheses of no effect. All experimental findings were reproducible.
Randomization	Mice were randomly allocated into groups.
Blinding	Some blind experiments were performed to avoid experimental bias. We confirmed that these data were not different from those obtained from regular experiments. Data sets were mainly not analyzed blindly, but some analysis were performed without knowledge of the group allocation. We confirmed that there was no difference in these analyzed results. Datasets were all processed according to uniform and identical processing steps. To maximize the objectivity, samples were treated according to the mouse ID number for in vivo experiments.

Reporting for specific materials, systems and methods

We require information from authors about some types of materials, experimental systems and methods used in many studies. Here, indicate whether each material, system or method listed is relevant to your study. If you are not sure if a list item applies to your research, read the appropriate section before selecting a response.

Materials & experimental systems

n/a	Involved in the study	
[ ]	[x]	Antibodies
[x]	[ ]	Eukaryotic cell lines
[x]	[ ]	Palaeontology and archaeology
[ ]	[x]	Animals and other organisms
[x]	[ ]	Human research participants
[x]	[ ]	Clinical data
[x]	[ ]	Dual use research of concern

Methods

n/a	Involved in the study	
[x]	[ ]	ChIP-seq
[x]	[ ]	Flow cytometry
[x]	[ ]	MRI-based neuroimaging

Antibodies

Antibodies used	The primary antibodies used were: anti-PLP (1:1 dilution; rat monoclonal, clone AA3 hybridoma supernatant, gift from Dr. Yamamura, Yamamura et al., J neurochem, 57, 1671-1680, 1991); anti-GFP (1:250; goat polyclonal, Rockland, Immunochemicals Inc., catalog# 600-101-215, lots# 34589); and anti-NG2 (1:500; rabbit polyclonal, Millipore, catalog# AB5320, lots# 3422864). The secondary antibodies used were: donkey anti-goat-IgG-Alexa488 (1:1000, Invitrogen, catalog# A32814, lot# VA293145); donkey anti-rabbit-IgG-Alexa488 (1:1000, Invitrogen, catalog# A21206, lot# 1480470); donkey anti-rat-IgG-Alexa 594 (1:1000, Invitrogen, catalog# A21209, lot# 1905801); donkey anti-rat-IgG-Alexa 647 (1:1000, abcam, catalog# ab150155, lot# GR3285492-1); and donkey anti-rabbit-IgG-Alexa 555 (1:1,000; Invitrogen, catalog# A31572, lot# 1945911). The antibodies used for in situ hybridization were: alkaline phosphatase-conjugated anti-DIG antibody (1:5000, Roche, catalog# 11093274910, lots# 32871922); and peroxidase-conjugated anti-FITC antibody (1:1000, Roche, catalog#11426346910, lot# 45220020).
Validation	Primary antibodies have been validated for use in the system under study in previous publications.<br>anti-PLP: Yamazaki et al. J. Neurosci. 39, 4036–4050 (2019).<br>anti-GFP: Tsutsui-Kimura et al. Curr. Biol. 27, 3042–3048.e4 (2017).<br>anti-NG2: Tanaka et al., Br. J. Pharmacol. 178, 1073–1094 (2021).

Animals and other organisms

Policy information about studies involving animals; ARRIVE guidelines recommended for reporting animal research

Laboratory animals	Details on the transgenic mice used in this study have been included in the Methods section of this manuscript. From 7- to 77-day-old male or female transgenic mice, C57BL/6 mice and 129SvEvTac mice were used.
Wild animals	The study did not involve wild animals.
Field-collected samples	The study did not involve sampled collected from the field.
Ethics oversight	All animal procedures were performed in accordance with the National Institutes of Health Guide for the Care and Use of Laboratory Animals and were approved by the Animal Research Committees of Yamagata University and Keio University.

Note that full information on the approval of the study protocol must also be provided in the manuscript.
